# The Structure of the Synaptic Vesicle-Plasma Membrane Interface Constrains SNARE Models of Rapid, Synchronous Exocytosis at Nerve Terminals

**DOI:** 10.3389/fnmol.2017.00048

**Published:** 2017-02-23

**Authors:** Cameron B. Gundersen

**Affiliations:** Department of Molecular and Medical Pharmacology, David Geffen UCLA School of MedicineLos Angeles, CA, USA

**Keywords:** synaptotagmin, active zone, docking filaments, tomographic reconstruction, transmitter release mechanisms, synapse function

## Abstract

Contemporary models of neurotransmitter release invoke direct or indirect interactions between the Ca^2+^ sensor, synaptotagmin and the incompletely zippered soluble, N-ethyl-maleimide-sensitive factor attachment protein receptor (SNARE) complex. However, recent electron microscopic (EM) investigations have raised pragmatic issues concerning the mechanism by which SNAREs trigger membrane fusion at nerve terminals. The first issue is related to the finding that the area of contact between a “fully primed” synaptic vesicle and the plasma membrane can exceed 600 nm^2^. Approximately four-thousands lipid molecules can inhabit this contact zone. Thus, renewed efforts will be needed to explain how the zippering of as few as two SNARE complexes mobilizes these lipids to achieve membrane fusion. The second issue emerges from the finding that “docking filaments” are sandwiched within the area of vesicle-plasma membrane contact. It is challenging to reconcile the location of these filaments with SNARE models of exocytosis. Instead, this commentary outlines how these data are more compatible with a model in which a cluster of synaptotagmins catalyzes exocytotic membrane fusion.

## Introduction

A major goal of neuroscience research is to clarify the molecular events that lead to the fast, synchronous release of neurotransmitters at chemical synapses. Seminal studies revealed that transmitter secretion is initiated via the depolarization-dependent entry of Ca^2+^ into the nerve terminal which triggers synaptic vesicle exocytosis (Katz, 1966; Heuser, 1989). This scenario raised two important questions: first, what is the identity of the target to which Ca^2+^ binds? and, second, how does Ca^2+^ binding to this target promote exocytosis? The consensus answer to the first question is that synaptotagmin 1 (or 2) is the physiological Ca^2+^-sensor for rapid, synchronous exocytosis at most nerve terminals (Südhof, 2014). The answer to the second question remains less clear. The prevailing view is that Ca^2+^-bound synaptotagmin triggers exocytosis by interacting directly or indirectly with soluble, N-ethyl-maleimide-sensitive factor attachment protein receptor (SNARE) proteins (Rothman, 2014; Südhof, 2014). However, as a follow-up to a comprehensive review (Meriney et al., 2014), this commentary will emphasize that much remains to be clarified about how SNARE proteins catalyze exocytotic membrane fusion. Additionally, it will be argued that recent empirical developments favor a simpler solution in which synaptotagmin is the template for exocytotic membrane fusion (Gundersen and Umbach, 2013).

## The Discovery of Synaptotagmin and SNAREs

Systematic efforts to clone and sequence the cDNAs encoding synaptic vesicle proteins led to the finding that a previously identified constituent of synaptic vesicles, p65, had two motifs that were related to presumptive Ca^2+^-binding domains of protein kinase C (Perin et al., 1990). These C2 domains were later shown to bind Ca^2+^ (Brose et al., 1992), and investigations from a number of groups ultimately led to the conclusion that synaptotagmins 1 and 2 were the principal Ca^2+^-sensors for synchronous exocytosis at chemical synapses (Südhof, 2014). The discovery of SNARE proteins was more convoluted. It began with the identification of soluble proteins (N-ethylmaleimide sensitive factor, or NSF, and the NSF adaptor proteins, or SNAPs) which were essential for membrane-trafficking in the Golgi apparatus. Then, because vertebrate brain had a high abundance of membrane targets for these soluble proteins, brain extracts were used in an affinity-purification scheme to identify the SNAP “receptors”, or SNAREs. The remarkable upshot of this effort was that the SNAREs were found to include a pair of plasma membrane-associated proteins (syntaxin A/B and synaptosome-associated protein of 25 kDa, or SNAP-25) and one synaptic vesicle protein (synaptobrevin 2; Söllner et al., 1993b). The further observations that SNAREs were targets of clostridial neurotoxins (Schiavo et al., 1992) and formed a ternary complex suggested that SNAREs might constitute a molecular link between a synaptic vesicle and the plasma membrane that could be exploited to drive membrane fusion (Söllner et al., 1993a,b). However, it was the finding that SNARE proteins formed parallel, rather than anti-parallel, complexes which supplied the conceptual basis for all subsequent models of SNARE involvement in membrane fusion (Hanson et al., 1997; Sutton et al., 1998). And, with the report that SNAREs promoted liposomal fusion (Weber et al., 1998), widespread efforts focused on the mechanism by which synaptotagmin interfaces with SNAREs to regulate exocytosis.

## Evolving Models of Synaptotagmin and SNARE Function in Synaptic Vesicle Exocytosis

**Figure 1:**
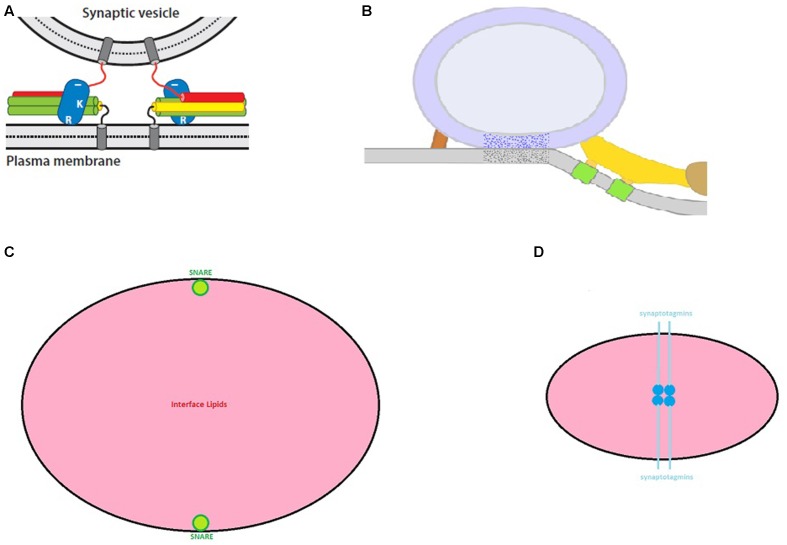
**Exocytosis models. (A)** In this class of SNARE model, release-ready synaptic vesicles are poised several nm from the plasma membrane with synaptotagmin (blue ovals) bound to partially zippered SNAREs (red, green and yellow cylinders) (from Rizo and Xu, 2015 with permission). **(B)** In this variant of SNARE model, the synaptic vesicle directly contacts the plasma membrane with SNAREs arrayed peripherally. Here, SNAREs presumably contribute to the ribs (yellow) and pins (red) (from Jung et al., 2016 with permission). **(C)** A “to-scale” illustration of SNARE membrane-spanning α-helixes (green) relative to the bulk lipid (pink) for a vesicle-plasma membrane contact area of 650 nm^2^. **(D)** As hypothesized in Gundersen and Umbach (2013), a quartet of synaptotagmins (deep blue: membrane-spanning helix; light blue: β-structure) should occupy the vesicle-plasma membrane interface (lipid in pink). Although this contact area was predicted to be 70–80 nm^2^ and could reach ~200 nm^2^, larger areas are incompatible with the dyad scheme. Nevertheless, the dyad model anticipates the presence of filaments (compare to Figure 2B) at this interface. For Figures 1, 2, the reader should consult the original article for details.

The crucial question to emerge from the preceding research was: “How does synaptotagmin control SNARE-mediated membrane fusion?”. The field still lacks a clear answer for this question. This absence of a unifying model of the exocytotic cascade has spawned a large number of competing proposals. Prominent examples of exocytotic models are given in the following publications: (Jahn and Fasshauer, 2012; Kasai et al., 2012; Mohrmann and Sørensen, 2012; Fang and Lindau, 2014; Kaeser and Regehr, 2014; Südhof, 2014; Rothman, 2014; Brewer et al., 2015; Rizo and Xu, 2015; Schneggenburger and Rosenmund, 2015; Zhou et al., 2015; Lou and Shin, 2016 and for a thorough critique of SNARE models see Meriney et al., 2014). With few exceptions, these models rely on the same three assumptions: The first is that SNARE complexes of suitably docked and primed synaptic vesicles are partially “zippered”. In other words, the coiled-coil interactions among synaptobrevin, syntaxin and SNAP-25 are arrested at an intermediate stage. The second assumption is that the completion of SNARE zippering supplies energy to drive the fusion of the vesicular and plasma membranes. The third assumption is that the Ca^2+^-bound state of synaptotagmin overrides the arrest of SNARE zippering to initiate the fusion process. Beyond these similarities, the reader should consult the cited references to understand how they differ in their treatment of auxiliary, SNARE-binding proteins (like, the complexins and the mammalian homologs of the nematode unc proteins, munc-13 and munc-18), and how they envision synaptotagmin relieving the arrest of SNARE zippering. However, for the purposes of this review, the most important difference among the cited models concerns their positioning of a release-ready synaptic vesicle. While some models locate the vesicle several nanometers from the plasma membrane (Figure 1A), others begin with the vesicular and plasma membranes in direct contact (Figure 1B). This difference in spatial organization has crucial implications as addressed next.

## Synaptic Vesicle Location Is a Crucial Consideration in Models of Nerve Terminal Exocytosis

SNARE-centric models of exocytosis typically begin with the architecture in Figures 1A,B. Figure 1A models are attractive, because it is intuitively evident how full zippering of the SNAREs might induce the formation of a fusion “neck” between the juxtaposed membranes. However, the paramount objection to such models is that they are incompatible with data from the vast majority of electron microscopic (EM) studies of nerve terminals. The following citations are culled from >30 articles which used serial reconstruction or EM tomography and found *no detectable separation* between the membrane of “docked” synaptic vesicles and the plasma membrane: (Schikorski and Stevens, 1997; Harlow et al., 2001; Xu-Friedman et al., 2001; Gustafsson et al., 2002; Rizzoli and Betz, 2004; Rostaing et al., 2006; Zampighi et al., 2006; Siksou et al., 2007; Nagwaney et al., 2009; Stigloher et al., 2011; Burette et al., 2012; Holderith et al., 2012; Leitinger et al., 2012; Marra et al., 2012; Szule et al., 2012; Watanabe et al., 2013; Cole et al., 2016; Jung et al., 2016). However, in defense of Figure 1A models, it was prominently noted (Fernández-Busnadiego et al., 2010) that vesicle-plasma membrane contacts were very infrequent in rat synaptosomes. Nevertheless, careful perusal of this article reveals that although such contacts were rare, they were still observed in unstimulated preparations. Thus, regardless of the appeal of Figure 1A models, they are not supported empirically. Instead, if SNAREs drive membrane fusion, synaptic vesicles need to be positioned as in Figure 1B. Before critiquing Figure 1B models, a detour will summarize important results from two recent investigations of the synaptic vesicle-plasma membrane interface.

**Figure 2:**
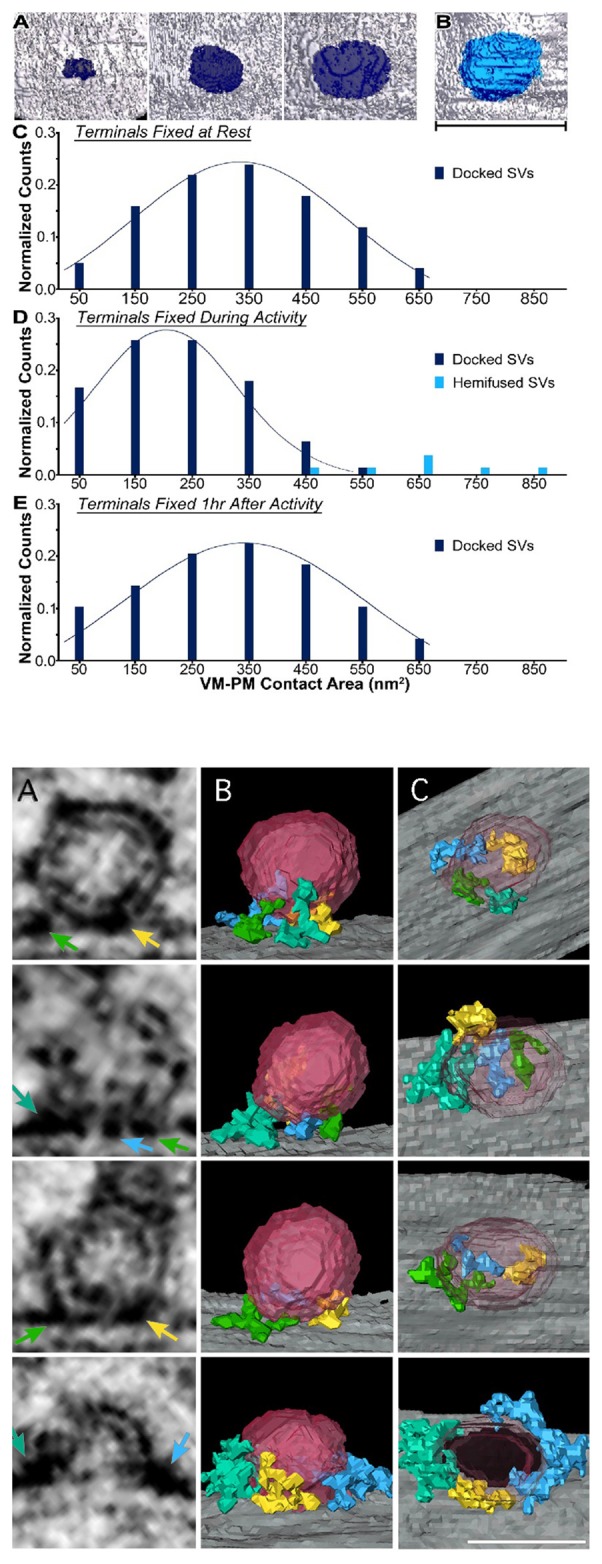
**Structural features of the synaptic vesicle-plasma membrane interface. (A)** These data are from Jung et al. (2016; with permission). In **(A)** are examples of contact areas between synaptic vesicles and the plasma membrane. **(B)** is a hemi-fused vesicle (scale bar: 50 nm). **(C–E)** are histograms of the vesicle contact areas for nerve terminals at rest, during nerve stimulation (10 Hz for 2 min) and 1 h after stimulation. **(B)** This figure (from Cole et al., 2016; with permission) shows how segmentation analysis identied filaments that project into the area of contact between synaptic vesicles and the plasma membrane. The colors of the arrows in the virtual sections (**A** panels) correspond to the filaments in the **(B)** panels and the images in the **(C)** panels have the vesicle removed to reveal the course of the filaments. Row 4 is a fusing vesicle. Scale bar: 35 nm.

First, Jung et al. (2016) measured the area of contact between docked vesicles and the plasma membrane for frog nerve terminals at rest, during and after activity (reproduced in Figure 2A). Their data indicated that the contact area reached 650 nm^2^ and was oval with average radii of ~12 and ~17 nm. Moreover, vesicles with large contact areas were depleted during synaptic activity (Figure 2A). This observation implied that vesicles with the largest contact areas were preferentially discharged in response to stimuli. This study also measured the thickness of the vesicular and plasma membranes away from the area of contact as well as within the contact zone. The result was that the aggregate thickness in the contact zone was twice the thickness of the individual membranes. The point here was that there was no detectable “sandwiching” of other material between the synaptic vesicle and the plasma membrane at their zone of contact. The other possibility was that any material that was “sandwiched” in this area did not measurably alter the thickness of the apposed membranes. Further implications of these results are addressed in Section “Pros and Cons of a Synaptotagmin-Only Model of Membrane Fusion”.

A second study of vesicle-plasma membrane contacts deployed segmentation analysis of tomographic images from freeze-substituted hippocampal neurons (Cole et al., 2016). Here, the provocative finding (reproduced in Figure 2B) was that “docking filaments” traversed the interface between docked synaptic vesicles and the plasma membrane. These filaments ranged from 3 nm to 8 nm in diameter and 10–47 nm in length. Although it was concluded that these filaments were likely to include SNAREpins (a term for SNARE complexes coined by Weber et al., 1998), variation in the filament shape and distributions in the renderings indicates some level of molecular heterogeneity in the composition of these elements. Clearly, it will be important empirically to establish the identity of these filaments.

SNARE-based models of exocytosis that begin with direct vesicle-plasma membrane contact (as in Figure 1B) are compatible with observations from myriad groups as well as the EM data in Figures 2A,B. However, if the quantitative results in Figure 2A generalize to other nerve endings, then Figure 1B models confront a significant practical challenge: based on the data of Jung et al. (2016) one can estimate the number of lipid molecules in a 650 nm^2^ membrane patch. By using the average cross sectional area of membrane phospholipids (0.65 nm^2^; Nagle and Tristram-Nagle, 2000), and ignoring the relatively high concentration of cholesterol in the synaptic vesicle membrane (Takamori et al., 2006), the four apposed hemi-bilayers comprising the zone of vesicle-plasma membrane contact harbor ~4000 lipid molecules. At the same time, empirical studies indicate that as few as two SNARE complexes support neuronal exocytosis (Sinha et al., 2011; in contrast, explicit models requiring 6–8 SNARE complexes have been presented: Jackson, 2010; Pantano and Montecucco, 2013). Given these parameters, the drawing in Figure 1C illustrates the challenge facing SNARE models: there is a sea of lipid flanked by two (to scale) membrane-spanning domains contributed by synaptobrevin or syntaxin. To date, no step-by-step model explains how these SNAREs perturb the intervening lipids to induce membrane fusion.

As an alternative to the situation illustrated in Figure 1C, it is worthwhile considering the possibility that SNARE complexes intrude into the area of contact between synaptic vesicles and the plasma membrane. As noted above, Jung et al. (2016) found no detectable thickening of membranes at this contact zone. Because EM images of SNARE complexes reveal 4 × 14 nm filaments (Hanson et al., 1997), there should have been a demonstrable thickening of this contact region, if SNAREs were sandwiched between these membranes. The other option is that SNAREs are buried in the hydrophobic interior of the opposed membranes. To countenance this explanation, one would need to accommodate the prominent surface charge of SNARE complexes (Sutton et al., 1998) within this apolar milieu. Although such a solution appears improbable, further investigation of the vesicle-plasma interface will be needed to clarify SNARE disposition and contributions to the fusion process.

As counterpoints to the models of Figures 1A,B, two other proposals were recently advanced. The first was based in part on the observations of Fernández-Busnadiego et al. (2010) that synaptic vesicles seldom contacted the plasma membrane but were frequently connected to it via filaments. It was suggested that these filaments corresponded to synaptotagmin which prevented SNAREs from zippering until Ca^2+^ entered the nerve ending (van den Bogaart et al., 2011). The primary argument against this model is the compelling evidence that release-ready synaptic vesicles directly contact the plasma membrane. The second model envisioned a ring of 16 synaptotagmins separating the vesicle from the plasma membrane and preventing full SNARE zippering (Wang et al., 2014). The concerns for this model are that the data of Jung et al. (2016) do not allow space for a synaptotagmin ring, and the filaments of Cole et al. (2016; see Figure 2B) are not symmetrical rings.

## Pros and Cons of a “Synaptotagmin-Only” Model of Membrane Fusion

The “dyad hypothesis” (Gundersen and Umbach, 2013) was advanced as an alternative to SNARE-based models of fast, synchronous exocytosis at nerve terminals. Briefly, it proposed that four synaptotagmins occupy the apical contact between a synaptic vesicle and the plasma membrane (Figure 1D). It further argued that Ca^2+^ binding by the C2 domains of these synaptotagmins leads to a lateral translocation of the membrane-spanning domains which serve as templates for membrane fusion. Although prominent features of this model remain to be tested empirically, the following discussion indicates where the dyad model is congruent with recent observations and where further investigation of features of synaptotagmins 1 and 2 is needed.

An explicit feature of the dyad model is that protein should be found spanning the vesicle-plasma membrane interface. In this respect, it is provisionally consistent with the observations of Cole et al. (2016) that macromolecules traverse this area. Moreover, because of relatively novel structural features (discussed in Gundersen and Umbach, 2013), synaptotagmins 1 and 2 can reside at this interface without changing the thickness of the membranes. Thus, the dyad scenario is compatible with the observation (Jung et al., 2016) that there is no thickening of the membranes where vesicles are docked. However, as discussed next, two important issues need to be resolved by future experiments.

The first issue is that material equivalent to the docking filaments reported by Cole et al. (2016) has not been detected at frog motor nerve terminals (Szule et al., 2012). The origin of this discrepancy will require further evaluation. The second issue is quantitative. Based on the proposed disposition of synaptotagmins at the vesicle-plasma membrane interface, the dyad model predicted 70–80 nm^2^ of direct contact between a synaptic vesicle and the plasma membrane. Even if one used a larger diameter for synaptotagmin’s membrane-spanning α-helix, and extended the length of the juxta-membrane β-strand to include the seven-residue polybasic region, this area still would compute to <210 nm^2^. In this respect, the dyad model confronts a quantitative challenge similar to SNARE models. Namely, how does one perturb a 650 nm^2^ area of vesicle-plasma membrane contact in a manner that is conducive to membrane fusion? To answer this query, it will be important to compare and contrast the area of direct contact between synaptic vesicles and the plasma membrane at other synapses to determine whether vesicles with >600 nm^2^ of contact are the norm. The results of such studies should help to clarify whether fusion is driven by SNAREs (as in Figure 1C), synaptotagmin (as in Figure 1D), or via a mechanism that has yet to be proposed.

## Conclusions

Advances in delineating the three dimensional organization and molecular composition of the synaptic vesicle-plasma membrane interface will be instrumental in distinguishing among current models of synaptic vesicle exocytosis. Although recent EM data (Figure 2) do not exclude SNAREs from catalyzing membrane fusion, the challenge embodied in Figure 1C will persist even if the area of lipid contact is halved. Instead, because synaptotagmins 1 and 2 can inhabit the synaptic vesicle-plasma membrane interface (as outlined in Gundersen and Umbach, 2013), it remains plausible that future studies will reveal a central role for synaptotagmin as a catalyst of “fast” membrane fusion.

## Author Contributions

This article was written by CBG.

## Funding

The author currently has no extramural or intramural funding.

## Conflict of Interest Statement

The author declares that the research was conducted in the absence of any commercial or financial relationships that could be construed as a potential conflict of interest.

## References

[B1] Brewer K. D. Bacaj T. Cavalli A. Camilloni C. Swarbrick J. D. Liu J.. (2015). Dynamic binding mode of a Synaptotagmin-1-SNARE complex in solution. Nat. Struct. Mol. Biol. 22, 555–564. 10.2210/pdb2n1t/pdb 26030874 PMC4496268

[B2] Brose N. Petrenko A. G. Südhof T. C. Jahn R. (1992). Synaptotagmin: a calcium sensor on the synaptic vesicle surface. Science 256, 1021–1025. 10.1126/science.1589771 1589771

[B3] Burette A. C. Lesperance T. Crum J. Martone M. Volkmann N. Ellisman M. H.. (2012). Electron tomographic analysis of synaptic ultrastructure. J. Comp. Neurol. 520, 2697–2711. 10.1002/cne.23067 22684938 PMC3856703

[B4] Cole A. A. Chen X. Reese T. S. (2016). A network of three types of filaments organizes synaptic vesicles for storage, mobilization, and docking. J. Neurosci. 36, 3222–3330. 10.1523/JNEUROSCI.2939-15.2016 26985032 PMC4792936

[B5] Fang Q. Lindau M. (2014). How could SNARE proteins open a fusion pore? Physiology 29, 278–285. 10.1152/physiol.00026.2013 24985331 PMC4103061

[B6] Fernández-Busnadiego R. Zuber B. Maurer U. E. Cyrklaff M. Baumeister W. Lucic V. (2010). Quantitative analysis of the native presynaptic cytomatrix by cryoelectron tomography. J. Cell Biol. 188, 145–156. 10.1083/jcb.200908082 20065095 PMC2812849

[B7] Gundersen C. B. Umbach J. A. (2013). Synaptotagmins 1 and 2 as mediators of rapid exocytosis at nerve terminals: the dyad hypothesis. J. Theor. Biol. 332, 149–160. 10.1016/j.jtbi.2013.04.029 23648184

[B8] Gustafsson J. S. Birinyi A. Crum J. Ellisman M. Brodin L. Shupliakov O. (2002). Ultrastructural organization of lamprey reticulospinal synapses in three dimensions. J. Comp. Neurol. 450, 167–182. 10.1002/cne.10310 12124761

[B9] Hanson P. I. Roth R. Morisaki H. Jahn R. Heuser J. E. (1997). Structure and conformational changes in NSF and its membrane receptor complexes visualized by quick-freeze/deep-etch electron microscopy. Cell 90, 523–535. 10.1016/s0092-8674(00)80512-7 9267032

[B10] Harlow M. L. Ress D. Stoschek A. Marshall R. M. McMahan U. J. (2001). The architecture of active zone material at the frog’s neuromuscular junction. Nature 409, 479–484. 10.1038/35054000 11206537

[B11] Heuser J. E. (1989). Review of electron microscopic evidence favouring vesicle exocytosis as the structural basis for quantal release during synaptic transmission. Q. J. Exp. Physiol. 74, 1051–1069. 10.1113/expphysiol.1989.sp003333 2560556

[B12] Holderith N. Lorincz A. Katona G. Rózsa B. Kulik A. Watanabe M.. (2012). Release probability of hippocampal glutamatergic terminals scales with the size of the active zone. Nat. Neurosci. 15, 988–997. 10.1038/nn.3137 22683683 PMC3386897

[B13] Jackson M. B. (2010). SNARE complex zipping as a driving force in the dilation of proteinaceous fusion pores. J. Membr. Biol. 235, 89–100. 10.1007/s00232-010-9258-1 20512644 PMC2944410

[B14] Jahn R. Fasshauer D. (2012). Molecular machines governing exocytosis of synaptic vesicles. Nature 490, 201–207. 10.1038/nature11320 23060190 PMC4461657

[B15] Jung J. H. Szule J. A. Marshall R. M. McMahan U. J. (2016). Variable priming of a docked synaptic vesicle. Proc. Natl. Acad. Sci. U S A 113, E1098–E1107. 10.1073/pnas.1523054113 26858418 PMC4776491

[B16] Kaeser P. S. Regehr W. G. (2014). Molecular mechanisms for synchronous, asynchronous, and spontaneous neurotransmitter release. Annu. Rev. Physiol. 76, 333–363. 10.1146/annurev-physiol-021113-170338 24274737 PMC4503208

[B17] Kasai H. Takahashi N. Tokamaru H. (2012). Distinct initial SNARE configurations underlying the diversity of exocytosis. Physiol. Rev. 92, 1915–1964. 10.1152/physrev.00007.2012 23073634

[B18] Katz B. (1966). Nerve, Muscle, and Synapse. New York, NY: McGraw-Hill.

[B19] Leitinger G. Masich S. Neumüller J. Pabst M. A. Pavelka M. Rind F. C.. (2012). Structural organization of the presynaptic density at identified synapses in the locust central nervous system. J. Comp. Neurol. 520, 384–400. 10.1002/cne.22744 21826661 PMC3263340

[B20] Lou X. Shin Y.-K. (2016). SNARE zippering. Biosci. Rep. 36:e00327. 10.1042/BSR20160004 27154457 PMC4859083

[B21] Marra V. Burden J. J. Thorpe J. R. Smith I. T. Smith S. L. Häusser M.. (2012). A preferentially segregated recycling vesicle pool of limited size supports neurotransmission in native central synapses. Neuron 76, 579–589. 10.1016/j.neuron.2012.08.042 23141069 PMC3526798

[B22] Meriney S. D. Umbach J. A. Gundersen C. B. (2014). Fast, Ca^2+^-dependent exocytosis at nerve terminals: shortcomings of SNARE-based models. Prog. Neurobiol. 121, 55–90. 10.1016/j.pneurobio.2014.07.001 25042638

[B23] Mohrmann R. Sørensen J. B. (2012). SNARE requirements *en route*to exocytosis: from many to few. J. Mol. Neurosci. 48, 387–394. 10.1007/s12031-012-9744-2 22427188

[B24] Nagle J. F. Tristram-Nagle S. (2000). Structure of lipid bilayers. Biochim. Biophys. Acta 1469, 159–195. 10.1016/S0304-4157(00)00016-2 11063882 PMC2747654

[B25] Nagwaney S. Harlow M. L. Jung J. H. Szule J. A. Ress D. Xu J.. (2009). Macromolecular connections of active zone material to docked synaptic vesicles and presynaptic membrane at neuromuscular junctions of mouse. J. Comp. Neurol. 513, 457–468. 10.1002/cne.21975 19226520 PMC4288958

[B26] Pantano S. Montecucco C. (2013). The blockade of the neurotransmitter release apparatus by botulinum neurotoxins. Cell. Mol. Life Sci. 71, 793–811. 10.1007/s00018-013-1380-7 23749048 PMC11113401

[B27] Perin M. S. Fried V. A. Mignery G. A. Jahn R. Südhof T. C. (1990). Phospholipid binding by a synaptic vesicle protein homologous to the regulatory region of protein kinase C. Nature 345, 260–263. 10.1038/345260a0 2333096

[B28] Rizo J. Xu J. (2015). The synaptic vesicle release machinery. Annu. Rev. Biophys. 44, 339–367. 10.1146/annurev-biophys-060414-034057 26098518

[B29] Rizzoli S. O. Betz W. J. (2004). The structural organization of the readily releasable pool of synaptic vesicles. Science 303, 2037–2039. 10.1126/science.1094682 15044806

[B30] Rostaing P. Real E. Siksou L. Lechaire J. P. Boudier T. Boeckers T. M.. (2006). Analysis of synaptic ultrastructure without fixative using high-pressure freezing and tomography. Eur. J. Neurosci. 24, 3463–3474. 10.1111/j.1460-9568.2006.05234.x 17229095

[B31] Rothman J. E. (2014). The principle of membrane fusion in the cell (Nobel lecture). Angew. Chem. Int. Ed Engl. 53, 12676–12694. 10.1002/anie.201402380 25087728

[B32] Schiavo G. Benfenati F. Poulain B. Rossetto O. Polverino de Laureto P. DasGupta B. R.. (1992). Tetanus and botulinum-B neurotoxins block neurotransmitter release by proteolytic cleavage of synaptobrevin. Nature 359, 832–835. 10.1038/359832a0 1331807

[B33] Schikorski T. Stevens C. F. (1997). Quantitative ultrastructural analysis of hippocampal excitatory synapses. J. Neurosci. 17, 5858–5867. 9221783 10.1523/JNEUROSCI.17-15-05858.1997 PMC6573206

[B34] Schneggenburger R. Rosenmund C. (2015). Molecular mechanisms governing Ca2+ regulation of evoked and spontaneous release. Nat. Neurosci. 18, 935–941. 10.1038/nn.4044 26108721

[B35] Siksou L. Rostaing P. Lechaire J. P. Boudier T. Ohtsuka T. Fejtová A.. (2007). Three-dimensional architecture of presynaptic terminal cytomatrix. J. Neurosci. 27, 6868–6877. 10.1523/JNEUROSCI.1773-07.2007 17596435 PMC6672225

[B36] Sinha R. Ahmed S. Jahn R. Klingauf J. (2011). Two synaptobrevin molecules are sufficient for vesicle fusion in central nervous system synapses. Proc. Natl. Acad. Sci. U S A 108, 14318–14323. 10.1073/pnas.1101818108 21844343 PMC3161593

[B37] Söllner T. Bennett M. K. Whiteheart S. W. Scheller R. H. Rothman J. E. (1993a). A protein assembly-disassembly pathway *in vitro* that may correspond to sequential steps of synaptic vesicle docking, activation, and fusion. Cell 75, 409–418. 10.1016/0092-8674(93)90376-2 8221884

[B38] Söllner T. Whiteheart S. W. Brunner M. Erdjument-Bromage H. Geromanos S. Tempst P.. (1993b). SNAP receptors implicated in vesicle targeting and fusion. Nature 362, 318–324. 10.1038/362318a0 8455717

[B39] Stigloher C. Zhan H. Zhen M. Richmond J. Bessereau J. L. (2011). The presynaptic dense projection of the *Caenorhabditis elegans* cholinergic neuromuscular junction localizes synaptic vesicles at the active zone through SYD-2/liprin and UNC-10/RIM-dependent interactions. J. Neurosci. 31, 4388–4396. 10.1523/JNEUROSCI.6164-10.2011 21430140 PMC3077722

[B40] Südhof T. C. (2014). The molecular machinery of neurotransmitter release (Nobel lecture). Angew. Chem. Int. Ed Engl. 53, 12696–12717. 10.1002/anie.201406359 25339369

[B41] Sutton R. B. Fasshauer D. Jahn R. Brunger A. T. (1998). Crystal structure of a SNARE complex involved in synaptic exocytosis at 2.4 A resolution. Nature 395, 347–353. 10.1038/26412 9759724

[B42] Szule J. A. Harlow M. L. Jung J. H. De-Miguel F. F. Marshall R. M. McMahan U. J. (2012). Regulation of synaptic vesicle docking by different classes of macromolecules in active zone material. PLoS One 7:e33333. 10.1371/journal.pone.0033333 22438915 PMC3306385

[B43] Takamori S. Holt M. Stenius K. Lemke E. A. Grønborg M. Riedel D.. (2006). Molecular anatomy of a trafficking organelle. Cell 127, 831–846. 10.1016/j.cell.2006.10.030 17110340

[B44] van den Bogaart G. Thutupalli S. Risselada J. H. Meyenberg K. Holt M. Riedel D.. (2011). Synaptotagmin-1 may be a distance regulator acting upstream of SNARE nucleation. Nat. Struct. Mol. Biol. 18, 805–812. 10.1038/nsmb.2061 21642968 PMC3130798

[B45] Wang J. Bello O. Auclair S. M. Wang J. Coleman J. Pincet F.. (2014). Calcium sensitive ring-like oligomers formed by synaptotagmin. Proc. Natl. Acad. Sci. U S A 111, 13966–13971. 10.1073/pnas.1415849111 25201968 PMC4183308

[B46] Watanabe S. Liu Q. Davis M. W. Hollopeter G. Thomas N. Jorgensen N. B.. (2013). Ultrafast endocytosis at *Caenorhabditis elegans* neuromuscular junctions. Elife 2:e00723. 10.7554/eLife.00723 24015355 PMC3762212

[B47] Weber T. Zemelman B. V. McNew J. A. Westermann B. Gmachl M. Parlati F.. (1998). SNAREpins: minimal machinery for membrane fusion. Cell 92, 759–772. 10.1016/s0092-8674(00)81404-x 9529252

[B48] Xu-Friedman M. A. Harris K. M. Regehr W. G. (2001). Three-dimensional comparison of ultrastructural characteristics at depressing and facilitating synapses onto cerebellar Purkinje cells. J. Neurosci. 21, 6666–6672. 11517256 10.1523/JNEUROSCI.21-17-06666.2001 PMC6763067

[B49] Zampighi G. A. Zampighi L. M. Fain N. Lanzavecchia S. Simon S. A. Wright E. M. (2006). Conical electron tomography of a chemical synapse: vesicles docked to the active zone are hemi-fused. Biophys. J. 91, 2910–2918. 10.1529/biophysj.106.084814 16877508 PMC1578491

[B50] Zhou Q. Lai Y. Bacaj T. Zhao M. Lyubimov A. Y. Uervirojnangkoorn M.. (2015). Architecture of the synaptotagmin-SNARE machinery for neuronal exocytosis. Nature 525, 62–67. 10.1038/nature14975 26280336 PMC4607316

